# Assessment of transient changes in oxygen diffusion of single red blood cells using a microfluidic analytical platform

**DOI:** 10.1038/s42003-021-01793-z

**Published:** 2021-03-02

**Authors:** Kevin Ziyang Chng, Yan Cheng Ng, Bumseok Namgung, Justin Kok Soon Tan, Soyeon Park, Sim Leng Tien, Hwa Liang Leo, Sangho Kim

**Affiliations:** 1 Department of Biomedical Engineering, National University of Singapore, Singapore, Singapore; 2 NUS Graduate School for Integrative Sciences and Efngineering, National University of Singapore, Singapore, Singapore; 3 Institute for Health Innovation & Technology, National University of Singapore, Singapore, Singapore; 4 Department of Hematology, Singapore General Hospital, Singapore, Singapore

**Keywords:** Isolation, separation and purification, Respiration

## Abstract

Red blood cells (RBCs) capability to deliver oxygen (O_2_) has been routinely measured by P50. Although this defines the ability of RBCs to carry O_2_ under equilibrium states, it cannot determine the efficacy of O_2_ delivery in dynamic blood flow. Here, we developed a microfluidic analytical platform (MAP) that isolates single RBCs for assessing transient changes in their O_2_ release rate. We found that in vivo (biological) and in vitro (blood storage) aging of RBC could lead to an increase in the O_2_ release rate, despite a decrease in P50. Rejuvenation of stored RBCs (Day 42), though increased the P50, failed to restore the O_2_ release rate to basal level (Day 0). The temporal dimension provided at the single-cell level by MAP could shed new insights into the dynamics of O_2_ delivery in both physiological and pathological conditions.

## Abstract (web summary)

Kevin Ziyang Chng et al. develop a microfluidic platform using a fluorescence quenching approach to assess transient changes in oxygen release rate of single red blood cells. They observe that biologically older red blood cells have a faster O_2_ release rate than younger cells and that storage of blood enhances this release rate.

## Introduction

Oxygen (O_2_) is required for maintaining various vital cellular functions and its supply to tissues is maintained by the cardiovascular system that ramifies throughout every organ in the body. Deprivation of O_2_ leads to tissue hypoxia, ultimately inducing cell dysfunction and apoptosis. Delivery of O_2_ is facilitated by red blood cells (RBCs) which constitute the majority of blood cells. Owing to its densely packed hemoglobin (Hb), RBCs can bind with O_2_ and release it on demand under deoxygenated conditions. Yet, the ability of RBCs to deliver O_2_ has been routinely characterized solely by their HbO_2_ affinity (P50), defined as the partial pressure of O_2_ (PO_2_) required to saturate Hb to 50% in a thermodynamic equilibrium process^1^. This metric, however, neglects the dynamic release rate of O_2_ from RBCs, and hence may be insufficient to assess the efficacy of RBC O_2_ delivery in the microcirculation. In contrast, assessing the temporal aspect of O_2_ delivery using the dynamic O_2_ release rate of RBCs would afford a more direct measure of the O_2_ transport by RBCs^2^.

O_2_ transport in the microvasculature typically occurs within a time frame of 4–7 s^3^. During the unloading process, O_2_ diffuses across the RBC membrane after dissociating from Hb. It has been shown that the internal cytoplasmic resistance of the RBC to O_2_ transport is not negligible^4,5^. Consequently, the time required for an RBC to unload O_2_ is dependent on both this internal resistance and the external milieu. Moreover, O_2_ uptake and release in RBCs are known to depend on their morphological and physiological properties^6^. Since RBCs exist as a non-homogenous entity of various biological ages with different biophysical and biochemical properties^7–9^, how these variations ultimately affect the O_2_ release rate remains unclear. Furthermore, a single-cell measurement is particularly important for RBC studies since there is vast cellular heterogeneity in the RBC population, which could result in the masking of functionally important subpopulations. Hence, profiling individual cells is essential to provide a representation of cellular level events in lieu of stochastic averages from bulk measurements^10^. Our motivation for this study is echoed by a recent study that investigated alterations in the O_2_ release rate of RBCs with varying cytoplasmic diffusivity^11^. Specifically, they have shown that an increase in pathlength or tortuosity of RBCs under engineered and diseased conditions can lead to a decrease in O_2_ transport. However, their measurements failed to isolate single cells from intercellular diffusion, unlike the current study.

Here, we use a diffusion parameter, D50, to better explain how fast each RBC can diffuse O_2_ to the surrounding tissues. For the isolation of a single RBC, we developed a microfluidic analytical platform (MAP) to examine the O_2_ diffusion from each single RBC, from which we derived the population variability of the O_2_ release dynamics. Using this MAP, we examined the effect of in vivo aging on the O_2_ release rate of RBCs and found that biologically older RBCs exhibited higher O_2_ release rates. We also demonstrated the effect of RBC storage lesion on the O_2_ release rate, which revealed a faster O_2_ release rate in stored RBCs than fresh cells. Moreover, we confirmed that the P50 value alone does not substantiate how fast RBCs can diffuse O_2_ even at the bulk level.

## Results

### MAP design and validation

**Fig. 1:**
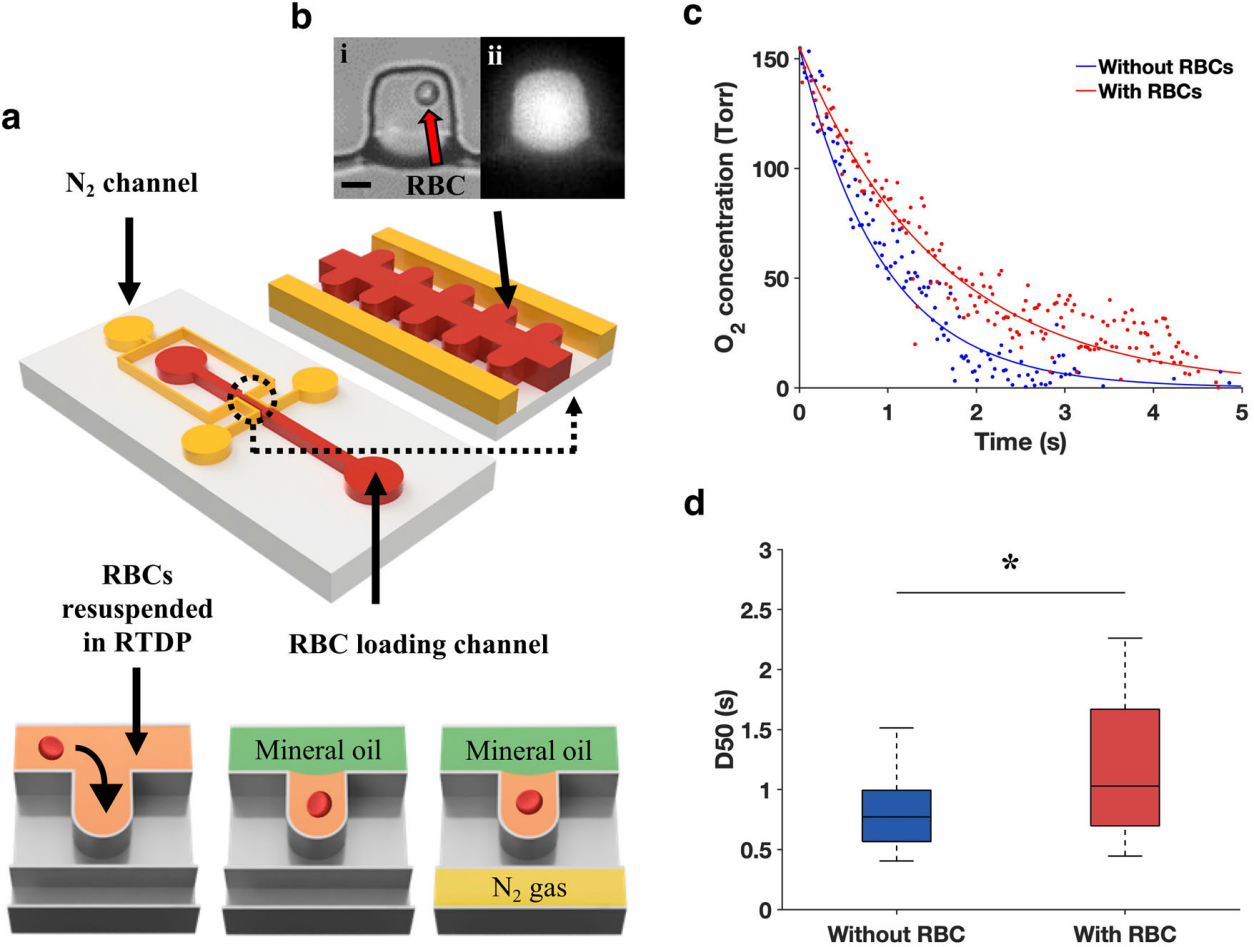
MAP to assess RBC O_2_ release rate at the single-cell level. **a** Schematic and working principle of the MAP. The microfluidic device consists of two fluid channels: RBC loading (red) and N_2_ flow (yellow). The RBC loading channel consists of an array of microwells for isolating single RBCs. A 25-µm PDMS wall separates the microwells from the N_2_ channels. The sample comprising RBCs suspended in tris(2,2′-bipyridyl) dichlororuthenium(II) hexahydrate (RTDP) solution was first loaded into the MAP. Mineral oil was then flushed into the microchannel to remove the cells in the main channel and isolate single RBCs in each microwell. N_2_ gas was then pumped into the gas channel to initiate the RBC deoxygenation process. **b** Trans- (i) and epi-illumination (ii) images of a microwell with a single RBC are shown. The red arrow indicates a single RBC isolated in the microwell. Scale bar = 10 µm. **c** Comparison of PO_2_ decay between microwells with (blue) and without (red) RBCs. The blue and red dots represent the raw experimental data while the corresponding solid lines represent the curves fitted using nonlinear regression. Blue line: ${{\mathrm{PO}}_{2}} = 155.3e^{ - 1.07t},\;({R}^{2} = 0.85)$. Red line: ${\rm{PO}_{2}} = 155.3e^{ - 0.63t},\;({R}^{2} = 0.86)$. **d** The decay constant obtained from the fitted curves was subsequently used to calculate the D50 (${\rm{D}}{50}= {\rm{ln}}{(2)}{\rm{K}}^{ - 1}$), with microwells loaded with RBCs (*n* = 82 of biologically independent RBCs) showing a significantly higher D50 (*P < 0.0001) than without RBCs (*n* = 127 of biologically independent RBCs). The central mark of the boxplot indicates the median, and the bottom and top edges of the box indicate the 25th and 75th percentiles, respectively. The whiskers extend to the most extreme data points not considered outliers.

The MAP was designed with an array of microwells for isolating single RBCs in the main microchannel. The microwells were then deoxygenated by nitrogen (N_2_) flowing in the parallel gas microchannel, which is separated from the microwells by 25-µm polydimethylsiloxane (PDMS) layer (Fig. 1a). Using fluorescence microscopy (Fig. 1b), we determined the rate of RBC deoxygenation from the temporal variations of fluorescence intensity in the microwells (Fig. 1c). As the presence of RBCs in the microwells provided an additional source of O_2_, higher rates of O_2_ release by the RBCs would thus reduce the rate of fluorescence decay in the microwells. This fluorescence quenching approach allows for the direct measurement of local O_2_ concentration. To validate the MAP, we established the decay rate of the O_2_ depletion in the microwells without RBCs, which corroborated the decay profile obtained from our computational simulation (*R*^2^ = 0.97) (Supplementary Fig. 2). To quantitatively assess the O_2_ release rate from single RBCs, we first fitted the experimental data using nonlinear regression, ${\rm{PO}}_2 = Ae^{ - Kt}$, where *A* is the initial PO_2_ in the microwell (155.3 mmHg), *K* denotes the decay constant, and *t* represents time. The obtained decay constant was then used to compute the time taken for the O_2_ level in the microwell to decrease by 50% from the initial equilibrium concentration (${\rm{D}}50 = {\rm{ln}}(2){\rm{K}}^{ - 1}$). Therefore, a higher D50 value indicates a faster O_2_ release rate from the RBC. Our results revealed that microwells with single RBCs showed a significantly larger D50 (1.17 ± 0.52 s) than those without RBCs (0.80 ± 0.29 s) (Fig. 1d).

### In vivo aging enhances the O_2_ release rate of RBCs

**Fig. 2:**
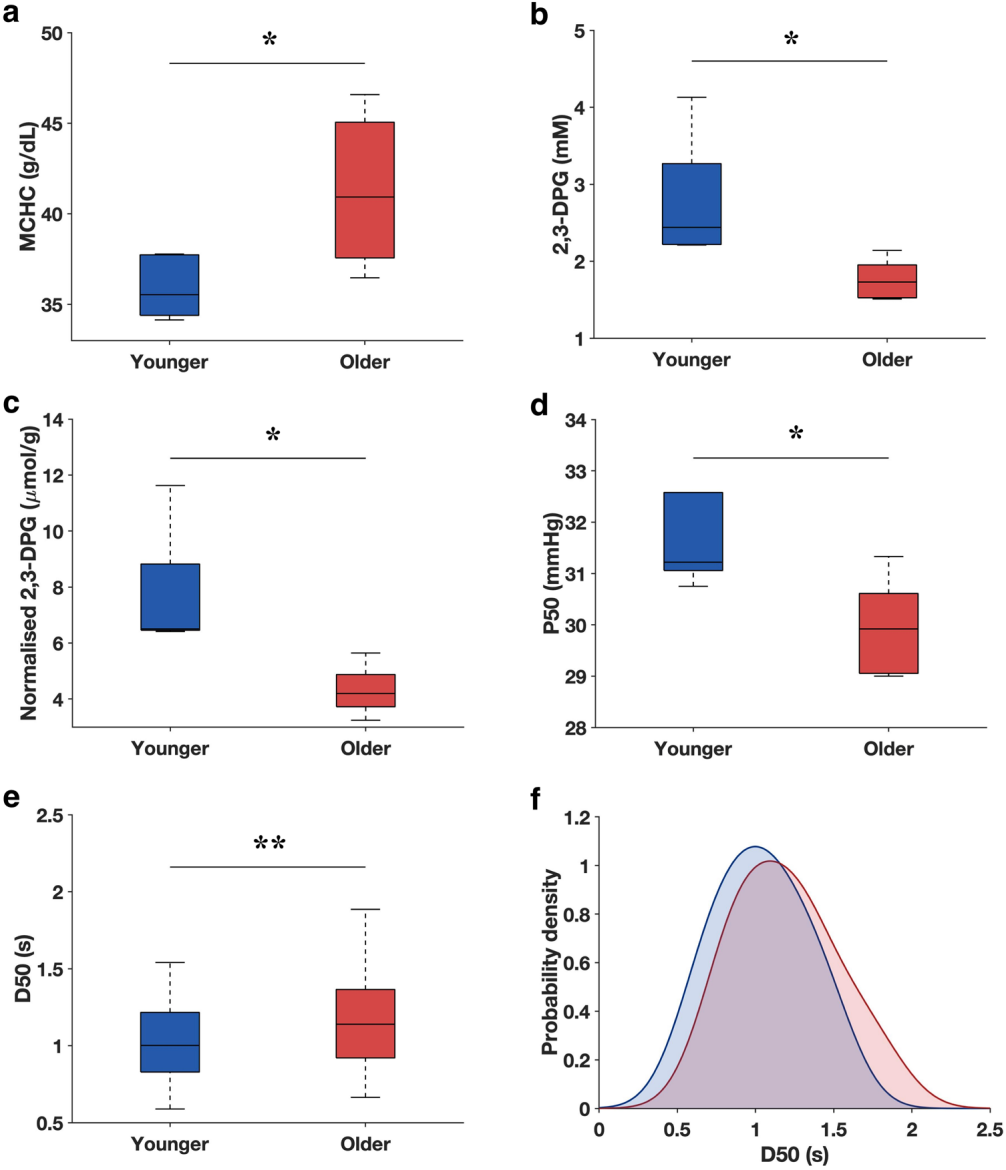
Effect of in vivo aging on the O_2_ affinity and release rate of RBCs. **a**–**d** MCHC, absolute 2,3-DPG concentration, normalized 2,3-DPG, and P50 of younger and older RBC samples (*n* = 5 of biologically independent samples). **e** D50 of younger and older RBCs. Older RBCs (*n* = 89 of biologically independent RBCs) showed a higher D50 than younger RBCs (*n* = 93 of biologically independent RBCs). **f** Probability density functions of D50 for younger (blue) and older (red) RBCs. It should be noted that results in (**a**–**d**) are based on bulk sample measurements, whereas those in (**e**, **f**) on single-cell measurements using the MAP. *P < 0.05, **P < 0.005. The central mark of the boxplot indicates the median, and the bottom and top edges of the box indicate the 25th and 75th percentiles, respectively. The whiskers extend to the most extreme data points not considered outliers.

To investigate the effects of in vivo aging on the O_2_ release rate of RBCs (Fig. 2), freshly drawn RBCs from healthy donors (*n* = 5) were density-fractionated into two groups: younger (top 20%) and older (bottom 20%). Older RBCs (41.3 ± 4.3 g/dL) had a significantly higher mean corpuscular hemoglobin concentration (MCHC) than younger RBCs (35.9 ± 1.7 g/dL). Absolute 2,3-DPG was also found to be substantially lower in older RBCs (1.76 ± 0.26 mM) than that in younger RBCs (2.80 ± 0.81 mM). As a result, normalized 2,3-DPG of older RBCs (4.32 ± 0.9 µmol/g Hb) was correspondingly lower than younger RBCs (7.78 ± 2.24 µmol/g Hb). Consequentially, the P50 of older RBCs (29.9 ± 1.0 mmHg) was lower than younger RBCs (32.0 ± 1.7 mmHg). The process of deoxygenation induces a conformational change in Hb from the relaxed (R) to the tense (T) structure, where Hb in the R state has a higher affinity for O_2_ than that in the T state. The 2,3-DPG binds to the central cavity of the deoxygenated Hb and anchors the molecular configuration of Hb in the T state. Hence, a lower 2,3-DPG level, as observed in the older RBCs, increases the Hb affinity for O_2_.

Interestingly, despite the increase in O_2_ affinity as determined by the P50 level (Fig. 2d), the diffusion rate (D50) of older RBCs (1.17 ± 0.32 s) was significantly higher than younger RBCs (1.03 ± 0.27 s) (Fig. 2e), implying a faster O_2_ release rate. D50 is governed by O_2_ diffusion through the cytoplasm and across the cell membrane. MCHC increases with RBC age through the reduction in cell volume via microvesiculation^12^. This results in a substantial tortuosity to the movement of solutes in the cytoplasm due to molecular crowding^13^. However, this is countered by a reduction in the diffusion path-length^2,14^. We observed an overall increase in D50 with RBC age, which implies the dominance of the reduced path-length over cytoplasmic diffusivity in regulating O_2_ diffusion. In probing the O_2_ release rates at the single-cell level, we were able to obtain and compare the distributions of D50 between older and younger RBCs (Fig. 2f), which showed that the full width at half maximum of the D50 distribution in older RBCs (0.75) was also larger than younger RBCs (0.64). In addition, the skewness for the D50 distributions of younger and older RBCs was determined to be 0.17 and 0.48, respectively, indicating a stronger deviation from normality toward higher D50 values in older RBCs.

### Blood storage enhances the O_2_ release rate of RBCs

**Fig. 3:**
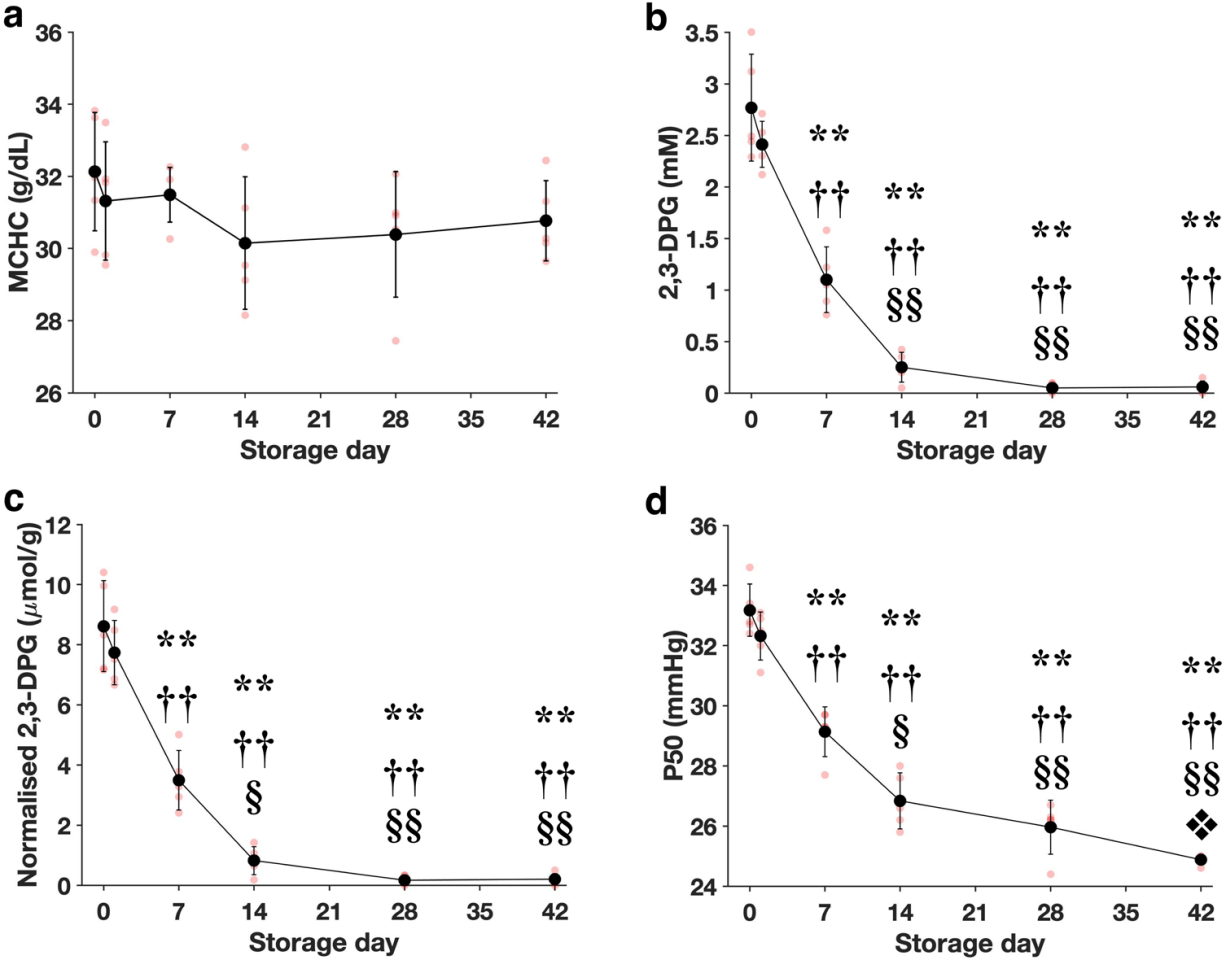
Effect of in vitro aging during blood storage on O_2_ affinity of RBCs. **a**–**d** MCHC, absolute 2,3-DPG concentration, normalized 2,3-DPG, and P50 during the storage period. All the results are based on bulk sample measurements (*n* = 5 of biologically independent samples). The significant difference relative to Day 0: *P < 0.05, **P < 0.0001. The significant difference relative to Day 1: ^†^*P* < 0.01, ^††^*P* < 0.0001. The significant difference relative to Day 7: ^§^*P* < 0.001, ^§§^*P* < 0.0001. The significant difference relative to Day 14: ^❖^*P* < 0.01. Error bars correspond to the standard deviation of the data.

To examine the effect of in vitro aging on the O_2_ release rate of RBCs, fresh RBCs were stored for 42 days following the standard blood banking protocol. Figure 3 shows changes in the O_2_ affinity-related factors of the stored RBCs. No significant alteration in the MCHC was observed during the storage duration. In contrast, absolute (Day 0: 2.77 ± 0.52 mM, Day 7: 1.10 ± 0.32 mM) and normalized 2,3-DPG concentrations (Day 0: 8.61 ± 1.51 µmol/g Hb, Day 7: 3.49 ± 0.99 µmol/g Hb) dropped drastically by Day 7 and came close to depletion on Days 28 and 42. Consequently, P50 of stored RBCs decreased significantly with storage duration, indicating an increase in O_2_ affinity (Day 0: 33.2 ± 0.87 mmHg, Day 42: 24.9 ± 0.16 mmHg) (Fig. 3d). Detailed experimental results can be found in Supplementary Table 1.

**Fig. 4:**
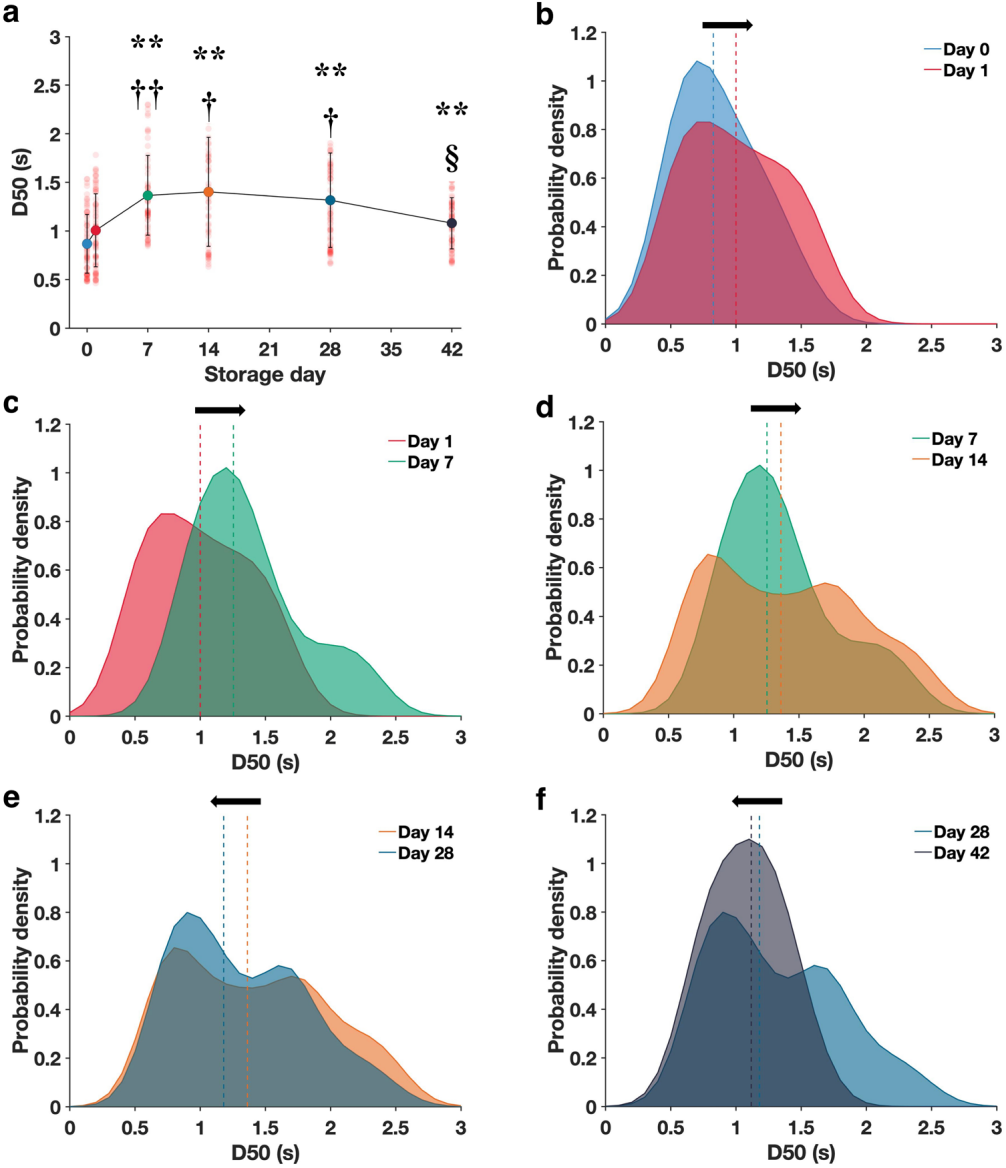
Effect of in vitro aging during blood storage on the D50 of RBC. **a** D50 changes during storage (Day 0: *n* = 72, Day 1: *n* = 71, Day 7: *n* = 60, Day 14: *n* = 55, Day 28: *n* = 75 and Day 42: *n* = 65 of biologically independent RBCs). **b**–**f** Probability distribution of D50 for each respective storage day: Day 0 (light blue), Day 1 (red), Day 7 (green), Day 14 (orange), Day 28 (dark blue), and Day 42 (black). Broken lines represent the median values of D50. The significant difference relative to Day 0: *P < 0.05, **P < 0.0001. The significant difference relative to Day 1: ^†^*P* < 0.001, ^††^*P* < 0.0001. The significant difference relative to Day 7: ^§^*P* < 0.001, ^§§^*P* < 0.0001. Error bars correspond to the standard deviation of the data.

As opposed to the decrease in the P50, the D50 of stored RBCs (Fig. 4) was significantly higher than that of the fresh RBCs (Day 0: 0.87 ± 0.30 s, Day 42: 1.08 ± 0.26 s). The D50 distribution at Day 0 assumed a Gaussian profile. However, the distribution deviated from Gaussian at Day 1 and tended toward a bimodal distribution by Day 14; this bi-modal distribution seemingly reverted to normality again by Day 42. Concomitantly, the distribution was initially right-shifted toward higher D50 but became left-shifted from Day 14. The full width half maximum (FWHM) for each of the storage durations was also calculated (Supplementary Fig. 3). We found that Day 14 (1.32) had an ∼86% increase in FWHM as compared to Day 0 (0.71). The FWHM then decreased after Day 14 and by Day 42, the FWHM reduced to ∼13% less than Day 0 (Day 28: 1.14, Day 42: 0.62). Considering these observations, we hypothesized that different RBC subpopulations undergo differential rates of hemolysis during storage, which would account for the emergence of bimodal distributions and gradual left-shift with longer storage durations. To confirm this, we measured the hemolysis level of density-fractionated stored RBCs (for 42 days) in a separate experiment. Notably, older (denser) RBCs (1.81 ± 0.36%) showed a ~35% higher hemolysis than younger RBCs (1.34 ± 0.39%) after 42 days.

### Rejuvenation of 2,3-DPG does not improve the O_2_ release rate

**Fig. 5:**
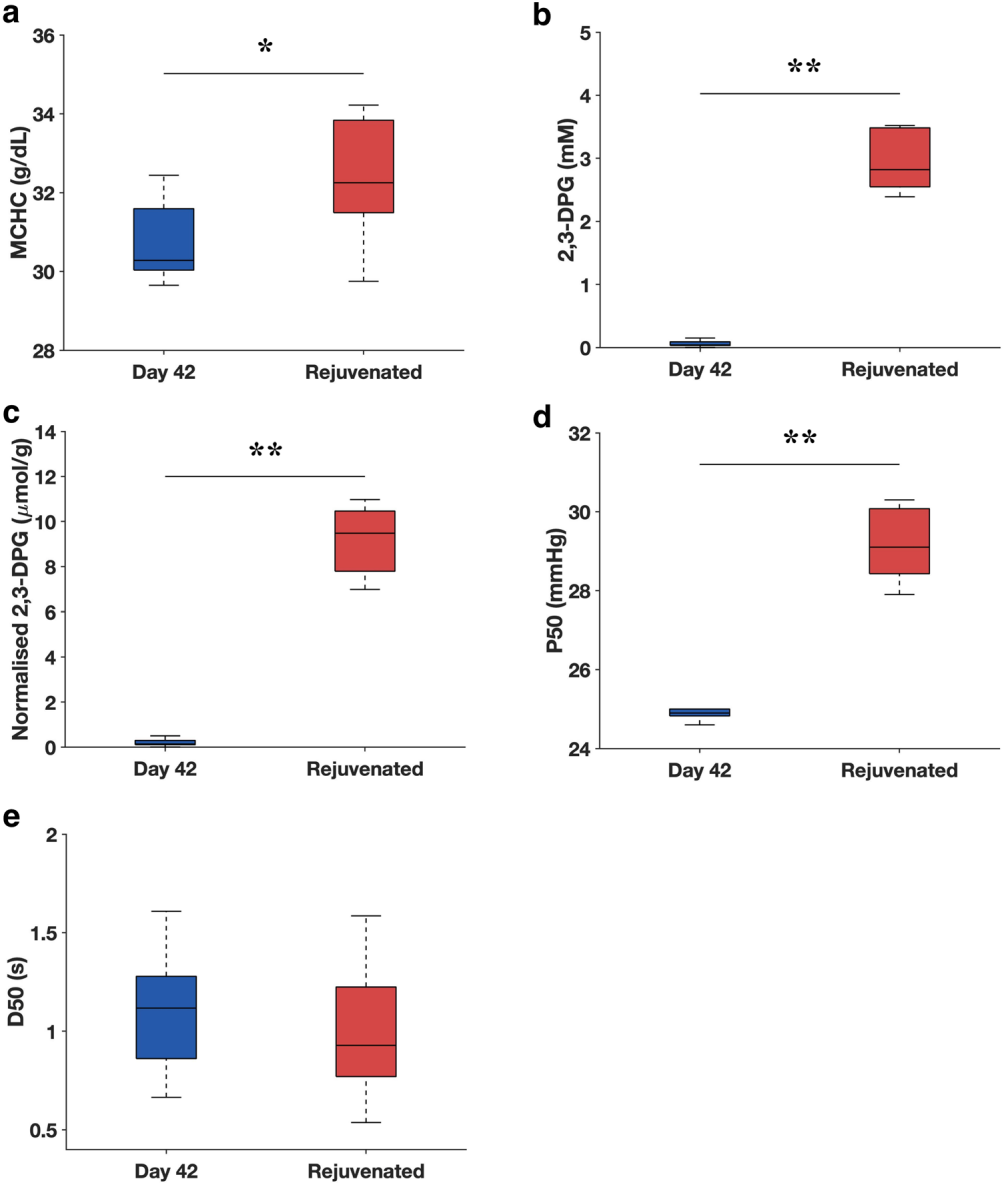
Effect of intracellular 2,3-DPG rejuvenation on the O_2_ release rate of RBCs stored for 42 days. **a**–**d** MCHC, absolute 2,3-DPG, normalized 2,3-DPG, and P50 before and after rejuvenation (*n* = 5 of biologically independent samples). **e** D50 of RBCs before (*n* = 65 of biologically independent RBCs) and after rejuvenation (*n* = 93 of biologically independent RBCs). *P < 0.05, **P < 0.001. The central mark of the boxplot indicates the median, and the bottom and top edges of the box indicate the 25th and 75th percentiles, respectively. The whiskers extend to the most extreme data points not considered outliers.

To determine the effect of 2,3-DPG rejuvenation on the O_2_ release rate of stored RBCs, Day 42 RBCs were incubated in 10 mM inosine and pyruvate solutions. The rejuvenation process increased the MCHC of the stored RBCs by ~5% (Day 42: 30.8 ± 1.1 g/dL, Rejuvenated RBCs: 32.4 ± 1.1 g/dL) (Fig. 5a). As expected, absolute and normalized 2,3-DPG concentrations of Day 42 RBCs were restored to the levels similar to those of fresh RBCs (Day 0: 2.77 ± 0.52 mM, Day 42: 0.06 ± 0.06 mM, Rejuvenated RBCs: 2.96 ± 0.51 mM for absolute values; Day 0: 8.61 ± 1.51 µmol/g Hb, Day 42: 0.20 ± 0.18 µmol/g Hb, Rejuvenated RBCs: 9.16 ± 1.63 µmol/g Hb for normalized values) (Fig. 5b, c). Rejuvenation of 2,3-DPG led to a significant increase (~20%) in the P50 of stored RBCs (Day 42: 24.9 ± 0.16 mmHg, Rejuvenated RBCs: 29.2 ± 0.99 mmHg) (Fig. 5d), although it was still significantly lower than the fresh RBCs (Day 0: 33.2 ± 0.87 mmHg). On the other hand, the measured D50 values (Fig. 5e) before and after rejuvenation were not significantly different (Day 42: 1.08 ± 0.26 s, Rejuvenated RBCs: 0.98 ± 0.28 s).

## Discussion

The fundamental role of RBCs is to deliver O_2_ to the tissues to sustain key metabolic processes that are required to maintain life. Hence, determining the efficacy of RBC O_2_ delivery is crucial in explaining a wide range of cellular phenomena in both physiological and pathological conditions. Many previous studies have emphasized the significance of the temporal aspect of O_2_ delivery^15,16^. Specifically, a decrease in RBC residence time or higher RBC flow velocity in the microcirculation could lead to tissue hypoxia due to reduced O_2_ release from the RBCs^17^.

The cellular heterogeneity that exists within the RBCs population demands single-cell analyses, which can provide new insights into RBCs O_2_ delivery functionally^18,19^. Di Caprio et al.^18^ developed a microfluidic platform to measure single-cell O_2_ saturation under continuous flow and demonstrated single-cell variability in the measured population. While the previous study provided steady-state measurements in terms of P50, this study focused on the measurement of dynamic temporal changes in isolated single RBCs. Recently, Richardson et al.^11^ also determined the O_2_ release rate by loading O_2_-sensitive fluorescent dyes into RBCs and discussed the relative contributions of various cellular properties to the overall release dynamics. In contrast, the present study localized the O_2_-sensitive fluorescent dye in the suspending medium to avoid disrupting the native state of the RBCs. Compared with the previous studies, a distinctive feature of our MAP is the array of microwells, which was designed to limit the diffusion to only between the suspending medium and the isolated single RBCs. Thus, the microwells effectively allow us to obtain the diffusion kinetics of the O_2_ unloading without interference from intercellular diffusion, along with the diffusion of the ambient gases into the suspending medium.

### O_2_ diffusion in RBCs

O_2_ uptake and release by RBCs involve complex molecular processes that occur over a span of length and time scales. RBCs uptake O_2_ in the alveoli in the lungs, within which O_2_ diffuses through blood plasma and the RBC cell membrane to reach the intracellular Hb. Within the RBCs, Hb-facilitated diffusion of O_2_ exhibits a biphasic trend with Hb concentration^20^. Moreover, increasing the amount of Hb molecules is likely to impose a substantial tortuosity to the movement of solutes in the cytoplasm^2^. This is supported by a previous study^11^ that showed a decrease in O_2_ transport rate across the cytoplasm due to an increase in MCHC. Nonetheless, our experimental results showed that older RBCs exhibited significantly higher O_2_ release rates (higher D50) than their younger counterparts. This suggests that within the physiological range of MCHC (i.e., younger: 35.9 ± 1.7 g/dl; older: 41.3 ± 4.3 g/dL), the concentration of Hb does not have a dominant effect on the O_2_ diffusion rate from RBCs.

During the aging process, whether in vivo or in vitro, RBCs experience a decline in the activities of vital metabolic enzymes and altered redox metabolism, leading to reduced ATP production^21^, oxidative damage to Band 3^22^ and membrane vesiculation^23,24^. Consequently, older RBCs experience a decrease in mean cell volume (MCV)^9,25^, which increases the MCHC, while reducing the path-length and the time required for O_2_ to diffuse across the cytoplasm to the inner side of cell membrane^2^, thus enhancing the rate of O_2_ release from RBCs^11^. The reductions in MCV and total surface area of the membrane in older RBCs could also lead to a decrease in the amount of membrane cholesterol^26–28^, which was previously shown to modulate O_2_ permeability in RBC membrane^29–33^. Therefore, these factors could cumulatively contribute to the elevation of D50 observed in older RBCs. While Richardson et al.^11^ demonstrated the effects of isolated changes in RBCs, our study suggests that there is a myriad of factors within the scheme of O_2_ diffusion of single RBCs. Concomitantly, diffusion of NO and CO_2_ could also be modulated through altered deoxygenation conditions^34,35^ and compromised Band 3^36,37^ as a consequence of the aging process. However, the effect of the two gases on our O_2_ measurements would be negligible since the RBCs are fully saturated with O_2_ and the NO produced by the NOS within the RBCs would be insignificant.

### 2,3-DPG

2,3-DPG is an intermediary metabolite in the Embden–Meyerhof glycolytic pathway that binds specifically to the central cavity of deoxyhemoglobin. This stabilizes the tense state of Hb, which reduces its O_2_ affinity as confirmed in our results (Figs. 2d and 3d)^38^. RBCs have a typical lifespan of 100–120 days during which they are constantly exposed to various insults from the external environment, leading to cumulative changes in their physical and chemical properties^39^. RBC senescence is associated with the exponential decline in glycolytic enzymes with RBC age, leading to reduced glycolysis and thus intracellular 2,3-DPG^40^. During in vitro blood storage, a similar decrease in glycolysis is attributed to the accumulation of lactic acid that lowers the pH of the storage medium^41^. While our results confirm that both in vivo (biological) and in vitro (storage), aging processes decrease the 2,3-DPG concentration and P50 of RBCs, we found no decline in their actual O_2_ diffusion rate. Interestingly, in both older and stored RBCs, D50 was significantly larger (O_2_ diffusion was faster) than in the younger (Fig. 2e) and fresh RBCs (Fig. 4), respectively. This suggests that 2,3-DPG does not play an important role in the regulation of O_2_ release from RBCs. While the decrease in 2,3-DPG concentration in aged RBCs enhances the Hb affinity for O_2_ (or reduces the P50 level), the release of O_2_ from Hb represents a subset of the whole O_2_ diffusion process. Hence, there might be other dominant factors that could lead to an increase in the O_2_ diffusion rate of aged RBC as determined by D50, which better reflects the totality of the O_2_ diffusion process. Moreover, the importance of 2,3-DPG on the whole scheme of O_2_ delivery remains unclear; a previous in vivo study demonstrated that the depletion of 2,3-DPG only had minor effects on the O_2_ reserve and O_2_ extraction^42^. Therefore, the use of both D50 (dynamic factor) and P50 (static factor) would provide a more comprehensive assessment of the O_2_ delivery capability of RBCs.

### Higher vs. lower D50

Oxygenated RBCs are constantly exposed to a large O_2_ gradient across the tissues before returning to the pulmonary circulation. Thus, it is likely that the intrinsic variability and distribution of D50 in RBCs are favorable in contributing to uniform O_2_ supply in the highly heterogeneous vascular networks. There exist various compensatory mechanisms in the cardiovascular system for regulating O_2_ delivery. For instance, a hypoxic environment could elicit a considerable increase in the cardiac output^43^. However, these mechanisms may be undermined under pathological conditions. Hence, RBCs with lower D50 could be better adapted to transport O_2_ to distal regions. We speculate that excessive variability in the distribution of D50, as shown with our blood storage study, may reduce the overall population O_2_ transport efficacy due to the detrimental contributions of the poorer functioning cells and may be explored as a potential indicator of hematological disorders. RBC distribution width (RDW), which measures the coefficient of variation of RBC size, has been associated with various deleterious clinical conditions such as higher mortality in older adults^44^, critical illnesses^45^, and increased odds of prevalent dementia^46^. Drawing a parallel between D50 and RDW, any major deviation from homeostatic distribution could have undesirable physiological consequences.

### Implication on the efficacy of blood transfusion

Transfusion of RBCs has been a stalwart therapeutic intervention for the treatment of acute and chronic anemia^47,48^. Its ultimate goal is to restore the tissue oxygenation level to maintain aerobic metabolism^49^. Currently, there is no consensus on the efficacy of stored RBC transfusion in restoring tissue oxygenation^50–52^. In this study, we found that the O_2_ diffusion rate of RBCs (D50) was enhanced with blood storage. RBCs undergo a myriad of biophysical and biochemical changes during storage, collectively defined as the storage lesion. Our P50 results show an increase in O_2_ affinity with storage duration that has mostly been interpreted as a potentially negative outcome. In contrast, the increase of D50 in stored RBCs suggests the apparent benefit of faster O_2_ supply to the tissues. RBC storage has been shown to increase the MCV of RBCs in some studies^53–58^, albeit marginally (<5%) especially when stored at 4 °C. Yet other studies showed no appreciable changes with storage^59–61^. These suggest that MCV changes with RBC storage have minimal impact on the O_2_ diffusion dynamics unlike in biological aging. Instead, we surmise that increased membrane permeability caused by oxidation and precipitation of membrane proteins and cellular dehydration (resulting from calcium influx and potassium efflux)^62,63^ may have contributed to the enhanced diffusion of solutes across the RBCs. However, it should be noted that this short-term, immediate increase in microvascular oxygenation might be offset by the more deleterious increase in hemolysis arising from the increased cellular mechanical fragility and impaired deformability^64^.

Various research groups have investigated the potential of prolonging the duration of stored blood beyond the 42 days period through rejuvenating techniques^65,66^. In this study, RBCs were rejuvenated after 42 days of storage with pyruvate and inosine to restore the depleted intracellular 2,3-DPG so as to investigate the potential effect on the altered O_2_ release rate. Our results show that the restoration of 2,3-DPG to basal levels (Day 0) has no significant effect on the O_2_ release rate. This is supported by a previous clinical study^67^ which showed no improvement in tissue oxygenation in hypoxic patients with increased 2,3-DPG concentrations in RBCs. Apart from the assessment of stored blood, we envision our proposed D50 metric to be used clinically to complement the existing measure of P50 in disease diagnosis, monitoring, or even intervention^1^. This may be especially pertinent in diseases associated with altered hemoglobin (such as sickle cell anemia^68^ and beta thalassemia^69^) or impaired perfusion (such as diabetic microangiopathies^70^ and neurodegeneration^71^). It is of note that our proposed D50 metric, similar to the conventional P50, does not consider the effects of RBC perfusion on the overarching tissue oxygenation efficacy. Aging of RBCs has been known to be associated with the reduction of cellular deformability^8^. The impaired RBC deformability may lead to reduced perfusion at the tissue level and this is well documented in diseases such as malaria^72^, sickle cells anemia^73^, and hereditary RBC membrane disorders^74^. This reduction in perfusion, corresponding to longer capillary transit time, could complement the augmented D50 in enhancing tissue O_2_ delivery, possibly as a compensatory mechanism to maintain PO_2_ homeostasis.

In summary, this paper presents a MAP for determining the O_2_ release rate of RBCs (D50) at the single-cell level. In addition to P50 to assess the RBC’s capability of delivering O_2_, we use D50 to complement our assessment of the dynamic process of O_2_ delivery. Interestingly, based on our D50 results, biologically older RBCs exhibited a faster O_2_ release rate than younger RBCs. We further demonstrated that blood storage enhanced the RBC O_2_ release and confirmed that 2,3-DPG rejuvenation had no significant impact on restoring the O_2_ release rate. The single-cell level data acquired with the MAP provides additional information (D50) that fills the knowledge gap in the O_2_ delivery of RBCs.

## Methods

### Blood collection and sample preparation

This study was approved by the National University of Singapore Institutional Review Board (H-18-007) and all procedures were performed in accordance with the approved guidelines. Healthy male volunteers aged between 21 and 50 years old, without any known pathological conditions that could affect the properties of the collected blood, were recruited. The number of samples in each experiment was derived by equally distributing the total number of participants recruited. Informed consent was obtained from all recruited participants. No data from research participants were excluded.

All blood collection and storage processes were performed under sterile conditions. Whole blood (20 mL) was collected from the donors into citrate-phosphate-dextrose (CPD) (Terumo) in the ratio of 7:1. The blood was then centrifuged at 2500*g* for 10 min at 4 °C (Sigma 2-6, Goettingen, Germany) to remove the plasma and buffy coat. The top 2-mm layer of the packed RBCs was also removed to minimize the presence of leukocytes. To examine the effect of in vivo aging, packed RBCs were further density fractionated at 4600*g* for 30 min at 4 °C to obtain younger (top 20% fraction) and older (bottom 20%) RBCs^75^. To examine the effect of storage duration, the packed RBCs were resuspended in saline–adenine–glucose–mannitol (SAGM) additive solution (Terumo) to a hematocrit of 50–70% and stored under standard blood bank conditions (2–6 °C). RBC samples were aliquoted aseptically for measurements on Days 0, 1, 7, 14, 28, and 42. To restore intracellular 2,3-DPG, RBCs were incubated in a 10 mM solution of pyruvate and inosine for 1 h at 37 °C^76^.

### Biochemical assay

MCHC and Hb were quantified using the direct cyanmethemoglobin^77^ with Drabkin’s reagent (D5941, Sigma), and the absorbance was recorded at 540 nm (Spark^®^, Tecan). Absolute 2,3-DPG was measured with a commercial kit (2,3-DPG, Roche). Normalized 2,3-DPG was obtained by normalizing the measured 2,3-DPG level by the corresponding MCHC from the same sample. Blood gases and pH were measured with a commercial blood gas analyzer (Epoc, Alere) and subsequently, the P50 values were determined^78^. Measurements for each sample were repeated successfully with similar results.

### Microfluidic analytical platform

The MAP was fabricated from PDMS (Dow Corning, MI) by standard soft lithography and replica molding techniques. The MAP consists of an array of microwells for trapping single RBCs, which are separated from two flanking gas microchannels by a thin PDMS wall (Fig. 1a). The detailed information of the channel design has been provided in Supplementary Fig. 1. On-chip O_2_ concentrations in the microwells were determined by an O_2_ sensitive probe, tris(2,2′-bipyridyl)dichlororuthenium(II) hexahydrate (RTDP) (544981, Sigma), dissolved in 1× phosphate-buffered saline (PBS) at 1 mg/mL (pH 7.2). The fluorescence intensity (*I*) was monitored using our microscope system (IX71, Olympus) under epi-fluorescence (U-MWB2, Olympus). The collisional quenching of this probe by O_2_ is described by the first-order Stern–Volmer equation: $I_0/I = 1 + K_q\left[ {{\rm{O}_{2}}} \right]$, where *I*_0_ is the intensity at 0% O_2_, *K*_*q*_ is the Stern–Volmer constant, and [O_2_] is the O_2_ concentration. The experimental *K*_*q*_ in our setup was determined as 2.2 × 10^−3^ µM^−1^, corresponding to previously reported values^79,80^.

RBC samples were washed with PBS twice before resuspending in RTDP at a hematocrit of 5%. The RBC suspension was then flowed into the MAP at 2 μL/min using a syringe pump (70-4504, Harvard Apparatus). After filling the microwells with RBCs, heavy mineral oil (330760, Sigma) was used to flush the residual RBCs and to isolate single RBCs in the microwell array. We introduced air flows in the two gas channels to initialize the O_2_ concentrations in the microwells to 100% saturation before starting the deoxygenation process. N_2_ gas was subsequently pumped through the gas channels under constant pressure (400 mbar) using a precision pressure controller (MCFS-EZ, Fluigent) to deoxygenate the microwells. Concurrently, the temporal variations of fluorescence intensity in the microwell were obtained from the fluorescent images (640 × 540 pixels, 4 × 4 binning) recorded with an sCMOS camera (Pco.edge, PCO AG, Kelheim, Germany) at 100 frame/s and a 40× objective (LCPlanFl, NA = 0.60, Olympus).

### Statistics and reproducibility

Statistical analyses were performed using a commercially available software package (Graphpad Prism 4). Results are presented as means ± SD. The Mann–Whitney test (two-tailed) was used to assess the statistical significance between microwells with (*n* = 82 of biologically independent RBCs) and without RBCs (*n* = 127 of biologically independent RBCs). Unpaired Student’s *t* tests (two-tailed) were used to assess the statistical difference between younger and older RBCs for MCHC, absolute 2,3-DPG, normalized 2,3-DPG, and P50 (*n* = 5 of biologically independent samples). Mann–Whitney tests (two-tailed) was used to assess the statistical significance between younger (*n* = 93 of biologically independent RBCs) and older (*n* = 89 of biologically independent RBCs) RBCs for D50. One-way ANOVA was used to assess the statistical significance across the storage duration for MCHC, absolute 2,3-DPG, normalized 2,3-DPG, and P50 (*n* = 5 of biologically independent samples). Kruskal–Wallis test was used to assess the statistical significance across the storage duration for D50 (Day 0: *n* = 72, Day 1: *n* = 71, Day 7: *n* = 60, Day 14: *n* = 55, Day 28: *n* = 75 and Day 42: *n* = 65 of biologically independent RBCs). When appropriate, post hoc analyses were performed with Tukey’s honestly significant difference test. Paired Student’s *t* test (two-tailed) was used to analyze the data before and after RBC rejuvenation for MCHC, absolute 2,3-DPG, normalized 2,3-DPG, and P50 (*n* = 5 of biologically independent samples). Wilcoxon test was used to analyze the data before (*n* = 65 of biologically independent RBCs) and after (*n* = 93 of biologically independent RBCs) RBC rejuvenation for D50. Single-cell distribution profiles were created using the kernel probability distribution. To describe the distribution profiles, full width at half maximum (FWHM = 2.355×*σ*) and skewness $(\frac{n}{{(n - 1)(n - 2)}}{\sum} {\left( {\frac{{x_j - \mu }}{\sigma }} \right)^3} )$ were used, where *µ* and *σ* represent the mean and SD, respectively, *n* is the total number of observations and *x*_*j*_ is the observation of *j*. The sample size for each experiment was reported in the figure caption. Data presentations were performed using MATLAB 2019a (MathWorks, USA).

### Reporting summary

Further information on research design is available in the Nature Research Reporting Summary linked to this article.

## Supplementary information

Supplementary Information

Description of Additional Supplementary Files

Supplementary Data 1

Reporting Summary

## Data Availability

All data are available in the main and supplementary files. Source data for the main figures can be found in Supplementary Data 1.

## Code availability

The in-house codes used for the analyses in this study are available from the corresponding author upon reasonable request.
