# Supplementary material for: Assessment of transient changes in oxygen diffusion of single red blood cells using a microfluidic analytical platform
Source: Commun Biol. 2021 Mar 2;4:271. doi: 10.1038/s42003-021-01793-z (PMC7925684; doi:10.1038/s42003-021-01793-z)
Supplement: Supplementary file 1 — Supplementary Information [file 42003_2021_1793_MOESM1_ESM.pdf]

Supplementary Information

Assessment of transient changes in oxygen diffusion of single red blood cells using a microfluidic analytical platform

Kevin Ziyang Chng[1], Yan Cheng Ng[1,2], Bumseok Namgung[1], Justin Kok Soon Tan[1], Soyeon Park[1,3], Sim Leng Tien[4], Hwa Liang Leo[1,2] and Sangho Kim[1,2,3,*]

[1]Department of Biomedical Engineering, National University of Singapore, Singapore

[2]NUS Graduate School for Integrative Sciences and Engineering, National University of Singapore, Singapore

[3]Institute for Health Innovation & Technology, National University of Singapore, Singapore

[4]Department of Hematology, Singapore General Hospital, Singapore

***Corresponding author**

Sangho Kim, PhD

Department of Biomedical Engineering, National University of Singapore

4 Engineering Drive 3, Block E4 #04-08

Singapore 117583

Phone: 65-6516 6713

Fax: 65-6872 3069

Email: bieks@nus.edu.sg

Supplementary Table 1 Consolidated experimental results (mean ± SD) for measured parameters of in vitro aging during blood storage.

Storage day	0	1	7	14	28	42	Rejuvenation
MCHC (g/dL)	32.13 ± 1.638	31.32 ± 1.639	31.49 ± 0.7555	30.15 ± 1.835	30.39 ± 1.742	30.77 ± 1.112	32.40 ± 1.745
Absolute 2,3-DPG (mM)	2.768 ± 0.5179	2.412 ± 0.2240	1.100 ± 0.3190	0.2500 ± 0.1430	0.0500 ± 0.04359	0.0600 ± 0.05612	2.960 ± 0.5118
Normalized 2,3-DPG (μmol/g Hb)	8.614 ± 1.513	7.741 ± 1.070	3.488 ± 0.9877	0.8198 ± 0.4660	0.1684 ± 0.1503	0.1973 ± 0.1848	9.159 ± 1.630
P50 (mmHg)	33.18 ± 0.8729	32.32 ± 0.8012	29.14 ± 0.8295	26.84 ± 0.9317	25.96 ± 0.8961	24.88 ± 0.1643	29.18 ± 0.9884
D50 (s)	0.8675 ± 0.3026	1.006 ± 0.3754	1.366 ± 0.4101	1.402 ± 0.5614	1.316 ± 0.4851	1.079 ± 0.2630	0.9847 ± 0.2778

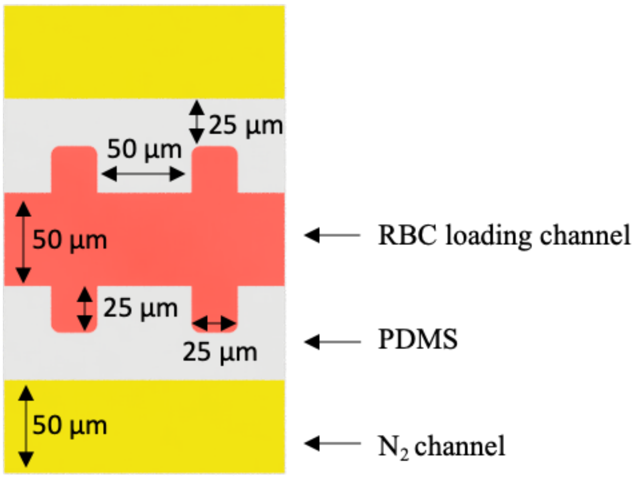

Supplementary Fig. 1 Dimension of MAP. RBCs loading channel (red) has a width of 50 µm with a series of 25 µm by 25 µm of microwells for isolating single RBCs. N_2 channel (yellow) has a width of 50 µm. A PDMS layer with a width of 25 µm separates the microwell and the N_2 channel. Height of both channels are 25 µm.

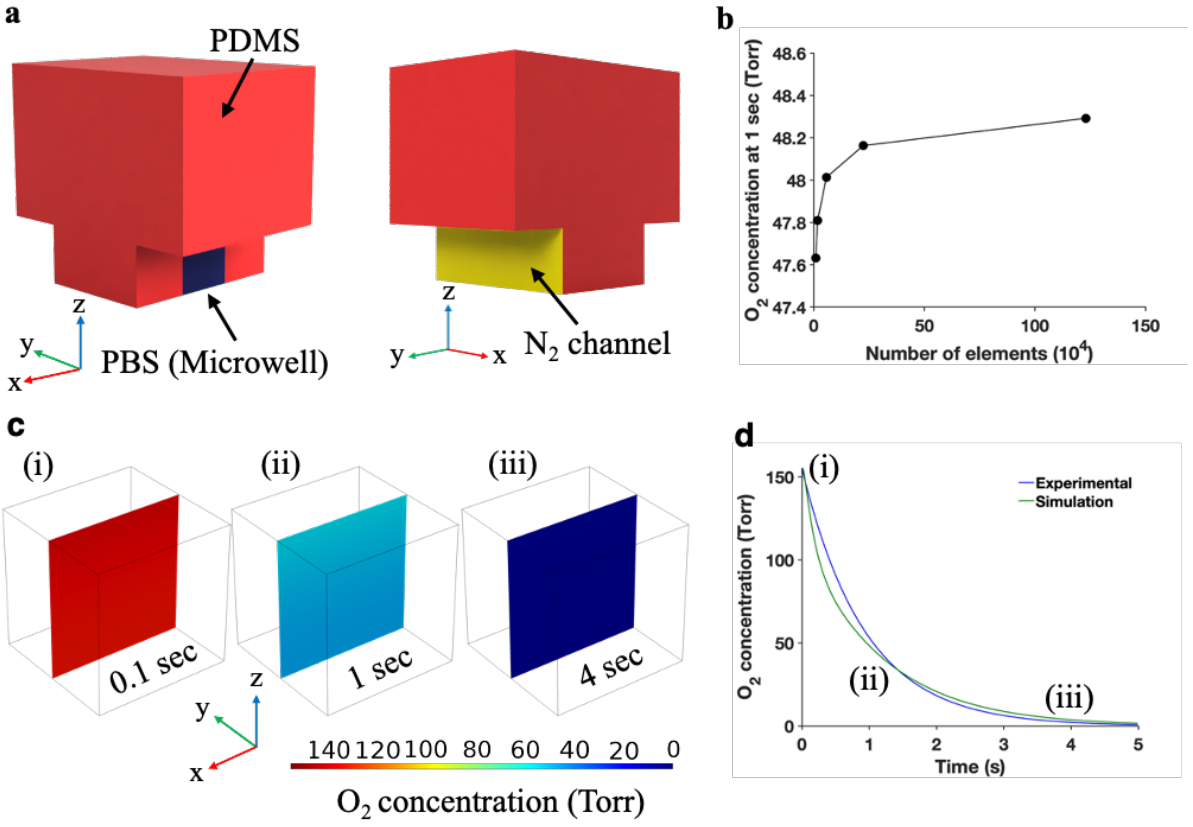

Supplementary Fig. 2 Computational simulation of the O_2 profile in MAP. **a** Transient 3-dimensional simulations of the O_2 concentration changes within the microwell were performed using COMSOL Multiphysics (Version. 5.3a, COMSOL Inc, MA, USA). Simulation domain consists of a single microwell filled with PBS (blue) bounded by PDMS (red). O_2 diffusion was governed by Fick's equation ${\partial C}/{\partial t} = D\nabla^2 C$, where C is the local O_2 concentration and D is the material-specific diffusion coefficient. For the simulation, the following parameters were used: initial O_2 tension for both PBS and PDMS = 155.28 Torr, diffusion coefficient of O_2 in PBS (D_{PBS}) = 1.9×10^{-9} m^2s, D_{PDMS}= 4.1×10^{-9} m^2s, solubility of O_2 in PBS = 0.218 $mMatm^{-1}$ and solubility of O_2 in PDMS = 1.62 mMatm[1]. The interface boundary condition between the PBS (microwell) and PDMS domains was calculated based on the stiff spring method in COMSOL, where the rate constant of O_2 from PBS to PDMS and from PDMS to PBS were 1.0×10^{-2} ms^{-1} and 1.7×10^{-3} ms^{-1} respectively. A Dirichlet boundary concentration of 0 mol/ m^3 was imposed on the interface between the PDMS layer and N_2 channel. For the remaining interfaces, no flux boundary conditions were imposed. Total simulation time was 5 s with a time-step of 0.01 s. **b** Grid independence was established through a mesh convergence test. **c** Cut plane plot of the O_2 concentration at the center of microwell at (i) time = 0.1 s, (ii) 1 s and (iii) 4 s. O_2 diffuses towards the N_2 channel (y-direction). **d** O_2 decay in the MAP without RBC (blue line) was consistent with the O_2 profile obtained from a computational simulation (green line) ($R^2 = 0.97$).

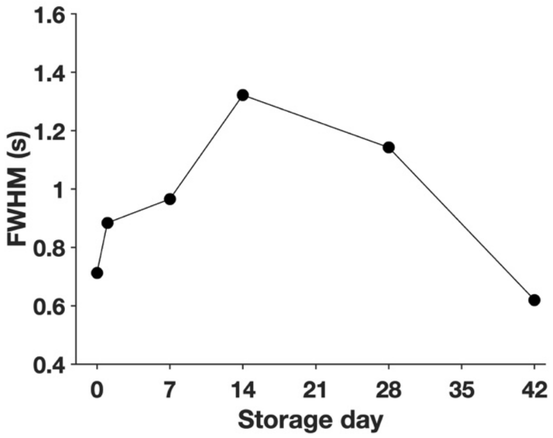

Supplementary Fig. 3 Full width half maximum (FWHM) for each storage day. FWHM of the D50 distribution increases from Day 0 to Day 14. Subsequently, it starts to decrease and by Day 42, the FWHM falls below the value on Day 0.
