# Supplementary material for: Assessment of transient changes in oxygen diffusion of single red blood cells using a microfluidic analytical platform
Source: Commun Biol. 2021 Mar 2;4:271. doi: 10.1038/s42003-021-01793-z (PMC7925684; doi:10.1038/s42003-021-01793-z)
Supplement: Supplementary file 2 — Description of Additional Supplementary Files [file 42003_2021_1793_MOESM2_ESM.pdf]

Description of Additional Supplementary Files

File Name: Supplementary Data 1

Description: Source data for the main figures.
