# Supplementary material for: Assessment of transient changes in oxygen diffusion of single red blood cells using a microfluidic analytical platform
Source: Commun Biol. 2021 Mar 2;4:271. doi: 10.1038/s42003-021-01793-z (PMC7925684; doi:10.1038/s42003-021-01793-z)
Supplement: Supplementary file 4 — Reporting Summary [file 42003_2021_1793_MOESM4_ESM.pdf]

nature research

Corresponding author(s): Sangho Kim

Last updated by author(s): 20/12/20

Reporting Summary

Nature Research wishes to improve the reproducibility of the work that we publish. This form provides structure for consistency and transparency in reporting. For further information on Nature Research policies, see our Editorial Policies and the Editorial Policy Checklist.

Statistics

For all statistical analyses, confirm that the following items are present in the figure legend, table legend, main text, or Methods section.

n/a	Confirmed	
☐	☒	The exact sample size (*n*) for each experimental group/condition, given as a discrete number and unit of measurement
☐	☒	A statement on whether measurements were taken from distinct samples or whether the same sample was measured repeatedly
☐	☒	The statistical test(s) used AND whether they are one- or two-sided<br>*Only common tests should be described solely by name; describe more complex techniques in the Methods section.*
☒	☐	A description of all covariates tested
☒	☐	A description of any assumptions or corrections, such as tests of normality and adjustment for multiple comparisons
☐	☒	A full description of the statistical parameters including central tendency (e.g. means) or other basic estimates (e.g. regression coefficient) AND variation (e.g. standard deviation) or associated estimates of uncertainty (e.g. confidence intervals)
☐	☒	For null hypothesis testing, the test statistic (e.g. *F*, *t*, *r*) with confidence intervals, effect sizes, degrees of freedom and *P* value noted<br>*Give P values as exact values whenever suitable.*
☒	☐	For Bayesian analysis, information on the choice of priors and Markov chain Monte Carlo settings
☒	☐	For hierarchical and complex designs, identification of the appropriate level for tests and full reporting of outcomes
☒	☐	Estimates of effect sizes (e.g. Cohen's *d*, Pearson's *r*), indicating how they were calculated

Our web collection on statistics for biologists contains articles on many of the points above.

Software and code

Policy information about availability of computer code

Data collection	PCO CamWare software for image acquisition. COMSOL Multiphysics (Version. 5.3a) for simulation raw data.
Data analysis	Images and raw data were quantified, analyzed and plotted in Matlab (R2020A). Statistical analysis was performed using Graphpad Prism 4.

For manuscripts utilizing custom algorithms or software that are central to the research but not yet described in published literature, software must be made available to editors and reviewers. We strongly encourage code deposition in a community repository (e.g. GitHub). See the Nature Research guidelines for submitting code & software for further information.

Data

Policy information about availability of data

All manuscripts must include a data availability statement. This statement should provide the following information, where applicable:

- Accession codes, unique identifiers, or web links for publicly available datasets
- A list of figures that have associated raw data
- A description of any restrictions on data availability

All source data that support the plots within this paper and other finding of this study are available from the provided supplementary data.

Field-specific reporting

Life sciences study design

All studies must disclose on these points even when the disclosure is negative.

Sample size	No sample size was calculated. Based on the total number of participants recruited, number of samples in each experiment was allocated equally among all the experimental conditions.
Data exclusions	No data from research participants were excluded.
Replication	For each experimental condition, measurements (MCHC, 2,3-DPG, P50 and D50) for each sample were repeated successfully with similar results.
Randomization	No specific randomization method was used, samples from participants were processed and allocated according to experimental timeline.
Blinding	No blinding was used.

Reporting for specific materials, systems and methods

We require information from authors about some types of materials, experimental systems and methods used in many studies. Here, indicate whether each material, system or method listed is relevant to your study. If you are not sure if a list item applies to your research, read the appropriate section before selecting a response.

Materials & experimental systems

n/a	Involved in the study	
☒	☐	Antibodies
☒	☐	Eukaryotic cell lines
☒	☐	Palaeontology and archaeology
☒	☐	Animals and other organisms
☐	☒	Human research participants
☒	☐	Clinical data
☒	☐	Dual use research of concern

Methods

n/a	Involved in the study	
☒	☐	ChIP-seq
☒	☐	Flow cytometry
☒	☐	MRI-based neuroimaging

Human research participants

Policy information about studies involving human research participants

Population characteristics	Healthy male adults aged between 21 and 50 years old, who are at least 50 kg, with no history of known pathophysiological conditions that can affect the characteristics of the collected blood.
Recruitment	Participants were recruited voluntarily via a recruitment email outlining above-mentioned prerequisite. Participants were enrolled without any bias selection.
Ethics oversight	National University of Singapore Institutional Review Board

Note that full information on the approval of the study protocol must also be provided in the manuscript.
